# Evaluation of a cone-beam computed tomography system calibrated for accurate radiotherapy dose calculation

**DOI:** 10.1016/j.phro.2024.100566

**Published:** 2024-02-29

**Authors:** Marta Bogowicz, Didier Lustermans, Vicki Trier Taasti, Colien Hazelaar, Frank Verhaegen, Gabriel Paiva Fonseca, Wouter van Elmpt

**Affiliations:** Department of Radiation Oncology (Maastro), GROW School for Oncology and Reproduction, Maastricht University Medical Centre+, Maastricht, The Netherlands

**Keywords:** Radiotherapy, Image quality, Cone-beam computed tomography, Dose calculation, CT number accuracy, Metal artifact reduction

## Abstract

**Background and purpose:**

Dose calculation on cone-beam computed tomography (CBCT) images has been less accurate than on computed tomography (CT) images due to lower image quality and discrepancies in CT numbers for CBCT. As increasing interest arises in offline and online re-planning, dose calculation accuracy was evaluated for a novel CBCT imager integrated into a ring gantry treatment machine.

**Materials and methods:**

The new CBCT system allowed fast image acquisition (5.9 s) by using new hardware, including a large-size flat panel detector, and incorporated image-processing algorithms with iterative reconstruction techniques, leading to accurate CT numbers allowing dose calculation. In this study, CBCT- and CT-based dose calculations were compared based on three anthropomorphic phantoms, after CBCT-to-mass-density calibration was performed. Six plans were created on the CT scans covering various target locations and complexities, followed by CBCT to CT registrations, copying of contours, and re-calculation of the plans on the CBCT scans. Dose-volume histogram metrics for target volumes and organs-at-risk (OARs) were evaluated, and global gamma analyses were performed.

**Results:**

Target coverage differences were consistently below 1.2 %, demonstrating the agreement between CT and re-calculated CBCT dose distributions. Differences in D_mean_ for OARs were below 0.5 Gy for all plans, except for three OARs, which were below 0.8 Gy (<1.1 %). All plans had a 3 %/1mm gamma pass rate > 97 %.

**Conclusions:**

This study demonstrated comparable results between dose calculations performed on CBCT and CT acquisitions. The new CBCT system with enhanced image quality and CT number accuracy opens possibilities for off-line and on-line re-planning.

## Highlights

• A novel cone-beam computed tomography system was evaluated for dose calculation.
• Accurate calibration of the cone-beam computed tomography was possible.
• Dose calculation was within 1% of the prescribed dose for complex treatments.
• This allows for direct offline and on-line re-planning using cone-beam imaging.

## Graphical abstract

**Figure:**
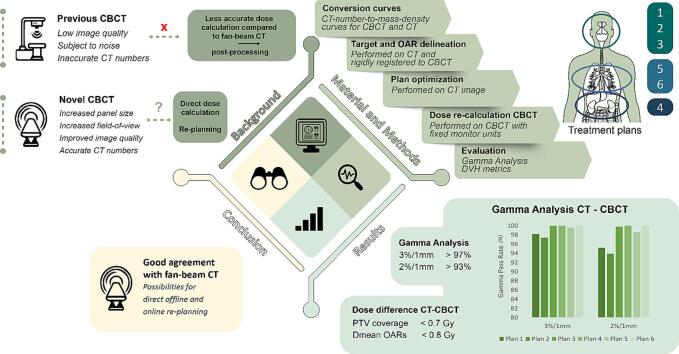

## 1 Introduction

The integration of cone-beam computed tomography (CBCT) on the radiotherapy treatment machine has brought significant progress to image-guided radiotherapy (IGRT) [1]. Recent developments in CBCT have significantly improved accurate patient positioning, and precise targeting, resulting in improved sparing of organs-at-risk (OARs) and overall treatment effectiveness [2], [3], [4]. Moreover, since anatomical variations in patients can occur, adaptive strategies are needed to account for discrepancies between planned and delivered dose. Therefore, there has been a growing interest in utilizing CBCT imaging for plan adaptation instead of repeat computed tomography (CT) [5], [6], [7], [8]. However, the lower image quality caused by photon scatter, limited field-of-view (FOV), and inconsistency in CT numbers for conventional CBCT compared to fan-beam CT can lead to lower dose calculation accuracy [2], [9], [10], [11], [12], [13], [14], [15].

In current linear accelerator (linac) based CBCT systems, image reconstruction is often carried out using filtered back projection with the Feldkamp-Davis-Kress (FDK) algorithm [10]. However, this approach has been found to handle image noise and artifacts insufficiently, and could lead to larger variations in CT numbers compared to fan-beam CT images [10], [16]. Dose calculations in photon therapy are typically based on a CT scan of the patient, with CT numbers converted to mass densities (MD) or relative electron densities (RED) by applying a conversion curve [17], [18]. This has been adapted to CBCT in multiple studies, but the noise and scatter considerably influence the relationship between the CT numbers and corresponding MD/RED [5], [9], [15], [16], [19], [20], [21], [22]. A study showed that for thoracic patients, the calibration method and accuracy were dependent on the CBCT system [23]. Other studies have explored the possibility of employing image post-processing to overcome the deviations in dose calculations due to differences in CT numbers for CBCT by overriding the MDs, modifying the CT numbers, using deformable image registration, or generating synthetic CT images (using deep learning networks) [9], [20], [24], [25], [26], [27], [28], [29], [30], [31], [32]. In recent years, iterative CBCT image reconstruction (iCBCT) techniques have been introduced and have been shown to enhance CT number accuracy and reduce noise levels compared to FDK [4], [10], [19], [33], [34]. The iCBCT is widely available for C-arm linacs and recently also for ring gantry linacs [35], [36], [37], [38]. The iCBCT has led to improved accuracy in dose calculation, although inconsistencies remain when dealing with different scattering conditions [5], [19].

Previous studies have focused on post-processing of CBCT images to remove the necessity for a repeat CT scan. More recent (commercial) developments in the hardware and software of CBCT systems have the potential to further improve dose calculation accuracy, without the need for image post-processing. It includes the integration of a larger kV imaging panel and image processing improvements, including scatter corrections enabling more accurate CT numbers as well as extended FOV (eFOV) and metal artifact reduction (MAR). However, for such new systems, it is important to perform a detailed evaluation of dose calculation accuracy before full integration into the clinical workflow.

This study aims to give a thorough evaluation of a new CBCT system by assessing the CBCT-based dose calculation performance, using commercially available phantoms representing different body and tumor sites. The dose distributions calculated on the CBCT images were compared to fan-beam CT to evaluate the possibility of using CBCT images for online and offline re-planning.

## 2 Material and methods

### 2.1 Phantoms

Three anthropomorphic phantoms were used for the evaluation of CBCT-based dose calculation, covering a variety of anatomical regions representing a wide range of typical target locations and complexities. The phantoms included a head phantom (CIRS Proton Therapy Dosimetry Head, Model 731-HN, Sun Nuclear, Norfolk, VA, USA), an abdominal phantom (CIRS Triple Modality 3D Abdominal Phantom, Model 057A, Sun Nuclear, Norfolk, VA, USA), and a thorax phantom (Alderson RANDO, Alderson Research Laboratories Inc., Long Island City, NY, USA). The head phantom was equipped with a titanium spine prosthesis attached by two metal screws at the C3 and C5 vertebrae, and a removable tooth, allowing for two scenarios, one tooth with standard composition and one containing a tungsten implant. This phantom was used to investigate MAR reconstruction. Six different treatment plans were created with the three phantoms.

### 2.2 Image acquisition

CBCT image acquisitions were performed using a novel HyperSight^TM^ CBCT imager (Varian Medical Systems, Palo Alto, CA, USA) integrated into a pre-clinical version of the Varian Halcyon treatment machine (v4.0, Varian Medical Systems). The HyperSight^TM^ CBCT ring gantry system comprised a large flat panel detector (86x43 cm^2^; larger than the predecessor system of which the dimensions are 43x43 cm^2^) with a cesium iodide scintillator with higher efficiency than the predecessor [39], and allowed for a scan reconstruction diameter of 53.8 cm. This system used an anti-scatter grid, with a 15:1 aspect ratio and 44 lamellae/cm. This detector enabled a pixel-size of 0.28 mm and frame rate of 70 frames per second allowing faster scan times than previous versions down to 5.9 s. Moreover, the acquisition was performed in full-fan mode while the predecessor ring gantry system scanned in half-fan mode. Additionally, the system was equipped with software for CBCT iterative reconstruction, which included techniques such as subject scatter corrections using the Acuros® CTS based scatter model [40], additional object scatter removal through the implementation of Monte-Carlo based hardware scatter correction, as well as MAR and eFOV (up to 70 cm). The system had two scanning modes, IGRT mode, and CBCTp mode with an image quality meant for treatment planning. In comparison to the IGRT mode, the CBCTp mode allowed for acquiring a topogram and more flexibility to vary the imaging parameters for the individual patients and to acquire higher quality images. Thus, the CBCTp mode showed more similarity to the standard CT imaging procedure. In this study, the CBCTp mode was used for all CBCT acquisitions with iterative reconstruction. Tube voltages used during acquisition were 125 kVp and 140 kVp. The CBCT acquisition and reconstruction parameters are summarized in Table 1.

**Table 1:** The upper part of the table lists the scan parameters for the CBCT acquisition of phantom 1 (CIRS Proton Therapy Dosimetry Head), phantom 2 (CIRS Triple Modality 3D Abdominal Phantom) and phantom 3 (Alderson RANDO). For phantom 1, two scans were acquired, one with the standard tooth (for plans 1 and 3), and one with the tungsten tooth (plan 2). The lower part of the table lists the plan parameters used in the individual plans. *Abbreviations*: MAR – metal artifact reduction; PTV – planning target volume; CW – clockwise rotation; MU – monitor unit.

**Treatment**	**Plan 1**	**Plan 2**	**Plan 3**	**Plan 4**	**Plan 5**	**Plan 6**
**Phantom**	Phantom 1	Phantom 1	Phantom 1	Phantom 2	Phantom 3	Phantom 3
**Note**		Metal tooth				
*Scan Parameters*						
**CBCT kVp**	125	125	125	140	125	125
**Slice thickness [mm]**	1	1	1	1	3	1
**CBCT field-of-view [mm]**	281	281	281	538	538	538
**CBCT pixel spacing [mm]**	0.55	0.55	0.55	1.05	1.05	1.05
**CBCT tube current time product [mAs]**	165	165	165	100	119	90
**CBCT CTDI_vol_ [mGy]**	41.5	41.5	41.5	25.7	13.3	10.1
**CBCT protocol**	Head & Neck	Head & Neck	Head & Neck	Pelvis Large	Thorax	Thorax
**CBCT acquisition time [s]**	5.9	5.9	5.9	5.9	5.9	5.9
**CT and CBCT reconstruction**	iterative	iterative	iterative	iterative	iterative	iterative
**MAR**	on	on	off	off	off	off
**Phantom dimension [cm]**	18 x 22 x 27	18 x 22 x 27	18 x 22 x 27	26 x 12.5 x 19	30 x 20 x 25	30 x 20 x 25
*Plan parameters*						
**Treatment site**	Head-and-neck	Head-and-neck	Brain	Liver	Lung	Lung
**Prescribed dose**	35x2 Gy to primary PTV 35x1.8 Gy to lymph nodes	35x2 Gy to primary PTV 35x1.8 Gy to lymph nodes	33x1.8 Gy	5x12 Gy	30x1.5 Gy	4x12 Gy
**Dose grid [mm^3^]**	2.5x2.5x3	2.5x2.5x3	1.25x1.25x2.5	1.25x1.25x2.5	2.5x2.5x3	1.25x1.25x2.5
**Number** **of arcs**	2	2	2	3	2	2
**Arc range** **CW [^o^]**	181–179	181–179	160–10	181–20	180.1–345	30–179.1
**Total** **MUs**	487.2	492.0	316.3	2227.3	322.1	2722.4

Fan-beam CT (SOMATOM Definition Drive; Siemens Healthineers, Forchheim, Germany) images were acquired according to clinical CT protocols varying per body site. Tube voltage was 120 kVp, and all reconstructions were performed with iterative reconstruction (ADMIRE level 3), a Qr40 filter and beam hardening correction (iBHC) for bone.

### 2.3 CT-number-to-mass-density conversion curve

The Gammex Advanced Electron Density phantom (Model 1472; Sun Nuclear, Middleton, WI, USA) was used to create a CT-number-to-mass-density conversion curve for dose calculation based on the CBCT images. The conversion curve specification followed the consensus guide for proton conversion curve specification and could be applied to photons [17]. Separate conversion curves were specified for each phantom size (small inner part and large full phantom) and tube voltage (125 kVp and 140 kVp). The same procedure was used to create conversion curves for the SOMATOM Drive CT scanner.

### 2.4 Treatment planning and dose re-calculation

**Fig. 1:**
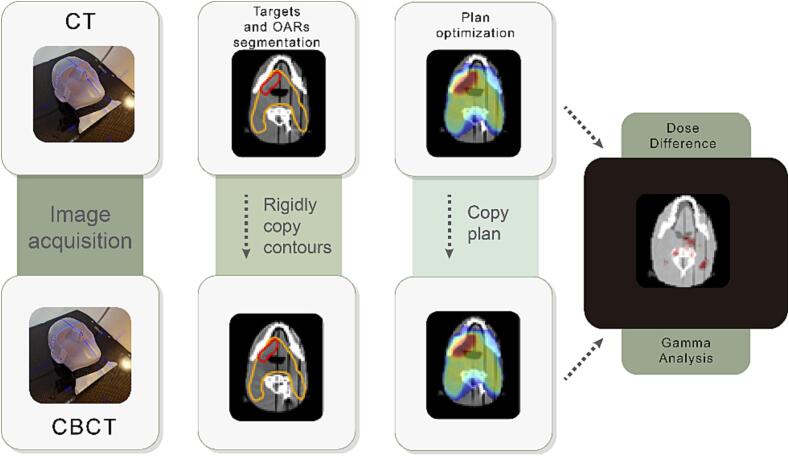
Overview of the steps performed in the dose calculation evaluation for both the fan-beam CT and CBCT images of the phantoms. A rigid registration was used to copy treatment plans from CT to CBCT. Dose difference was quantified based on dose delivered to targets and organs at risks and using gamma analysis.

Planning target volumes (PTVs) and OARs were delineated on the CT images and copied to the CBCT images following rigid image registration. The plans were optimized based on the CT images in Eclipse (v17, Varian Medical Systems) treatment planning system (TPS) using the Acuros dose calculation algorithm [41]. In the six plans, different targets were simulated with different levels of heterogeneity in surrounding tissues: Plan 1 and 2: head-and-neck cancer; Plan 3: brain tumor; Plan 4: liver metastasis; Plan 5: advanced stage (small cell) lung cancer; Plan 6: early stage lung cancer. This translated into a variety of planning techniques with different levels of complexity: simultaneous integrated boost (Plan 1 and 2), standard fractionation (Plan 3 and 5), and stereotactic treatment (Plan 4 and 6). Two head-and-neck plans were created to evaluate the influence of potential metal artifacts caused by metal implants on the accuracy of dose distribution. In the scan used for Plan 1, only the prosthesis was present, while in the scan used for Plan 2, the tungsten tooth was inserted. Detailed information about planning settings, can be found in Table 1. Target and OAR contours are shown in the Supplementary Materials (Supplement S1). Finally, these plans were copied to the CBCT images and the dose was re-calculated using the same TPS and with the same number of monitor units. The workflow is illustrated in Fig. 1.

### 2.5 Dose metric evaluation

To provide information about the clinical significance of the observed dose differences, dose-volume-histogram (DVH) metrics for the PTVs and relevant OARs were compared. For the PTVs, the metrics included the PTV coverage difference based on D_99%_ (Plan 3 and 4), D_98%_ (Plan 1 and 2), or D_95%_ (Plan 5 and 6) as well as the difference in mean (D_mean_) and near maximum (D_0.03cc_) dose. Depending on the OAR type, the mean or maximum (D_max_) dose difference was reported. The dose differences were calculated with CT as reference (ΔD = D_CBCT_ – D_CT_).

Furthermore, gamma analysis was performed in python v3.8 using the pymedphys (v 0.39.2) library [42]. The dose distributions optimized on the CT and re-calculated on CBCT were exported from the TPS and the dose grids were aligned based on the plan’s isocenter position. The dose distribution calculated on CT was used as reference and compared to the CBCT dose calculation using global gamma analysis with criteria of 3 %/1mm and 2 %/1mm. A lower dose threshold of 20 % of the prescribed dose was used.

## 3 Results

**Fig. 2:**
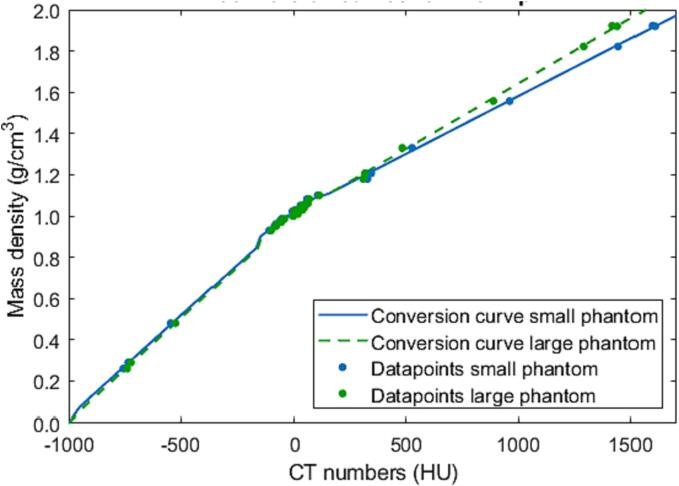
CT-number-to-mass-density conversion curve (Hounsfield look-up table; HLUT) generated for the small (head-sized) phantom (blue line) and large (body-sized) phantom (green dashed line) for 125 kVp for the CBCT scanner (the curves for the 140 kVp spectrum can be found in Fig. S6 in the supplementary materials along with the data used to create the curves).

The CT numbers of the CBCT images of the calibration phantom followed the trend expected from fan-beam CT, with the CT number for water close to 0 HU (-8 HU and −1 at 125 kVp for the small and large phantom respectively), which allowed for generating a CT-number-to-mass-density conversion curve following the step-by-step guide for fan-beam CT conversion curve specification shown in Fig. 2. The CT number size dependency (beam hardening) resulted in small deviations between these two curves, and therefore the appropriate conversion curve for each body site (plan 1–3: small phantom curve, plan 4–6: large phantom curve) was applied. A detailed description of the generation and accuracy of the conversion curves is given in Supplement S2.

**Fig. 3:**
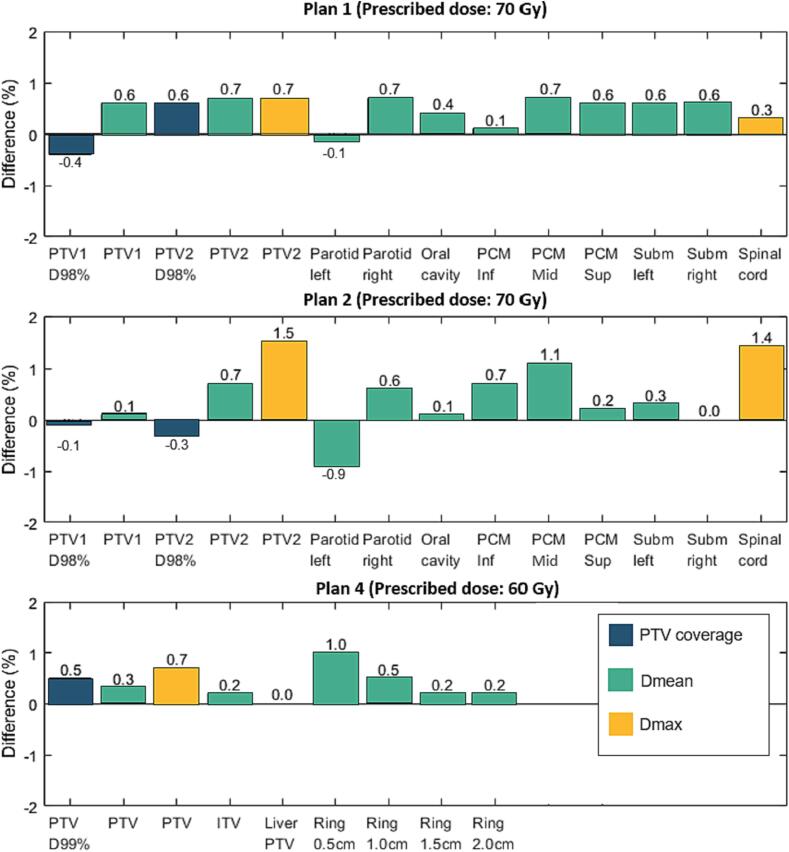
Bar plots for dose differences (percentage of prescribed dose) in the evaluated DVH metrics between dose calculation on a fan-beam CT and CBCT (D_CBCT_ – D_CT_) for plans 1, 2 and 4. The PTV coverage (blue bars) is quantified by D_98%_ (Plan 1 and 2) and D_99%_ (Plan 4). (Dose differences in Plan 3, 5 and 6 are shown in Fig. S8 in the supplementary materials). *Abbreviations:* PTV – planning target volume; PCM – pharyngeal constrictor muscle; Inf – inferior, Mid – middle, Sup – superior; Subm – Submandibular.

The target coverage differences were below 1.2 % of the prescribed dose (Fig. 3, Fig. S8, and Table S4). In all plans, a PTV D_mean_ difference (CBCT – CT) below 0.5 Gy (<0.7 % of the prescribed dose) was observed, with a slight PTV D_mean_ over-estimation in CBCT-based dose calculation. The differences in PTV coverage were also below 0.5 Gy for the majority of the plans, with the exception of Plan 3, where CBCT-based PTV coverage dropped by 0.7 Gy (1.2 %) in comparison to the CT-based calculation. Visual plan inspection revealed the under-dosage region was on the uppermost PTV slice. PTV D_0.03cc_ showed an agreement with differences below 0.7 %, apart from Plan 2 where 1.1 Gy (1.5 %) dose over-estimation was observed. The hotspot was in a region of remaining streak artifacts in the CBCT image a few slices above the tooth implant.

The D_max_ in the analyzed OARs were in general over-estimated in the CBCT-based in comparison to CT-based dose calculations. Nevertheless, the D_max_ difference remained below 0.5 Gy in most cases, except for the spinal cord in Plan 2 with a difference of 1.0 Gy (1.4 %) located in proximity to the titanium spine implant, and the brainstem in Plan 3 with a difference of 1.1 Gy (1.9 %) located in high dose gradient close to the PTV. Moreover, the re-calculation of Plans 1, 3, 5, and 6 demonstrated that the D_mean_ differences for the OARs were consistently less than 0.5 Gy, corresponding to less than 0.8 % of the prescription dose. In Plan 2, slightly higher discrepancies were found between the CBCT-based and the CT-based dose calculation, with a D_mean_ under-estimation of 0.6 Gy (0.9 %) for the left parotid and an over-estimation of 0.8 Gy (1.1 %) for the middle pharyngeal constrictor muscle. Larger differences were also observed for Plan 4, where D_mean_ was over-estimated by 0.6 Gy (1.0 %) for a 0.5 cm ring around the PTV.

**Fig. 4:**
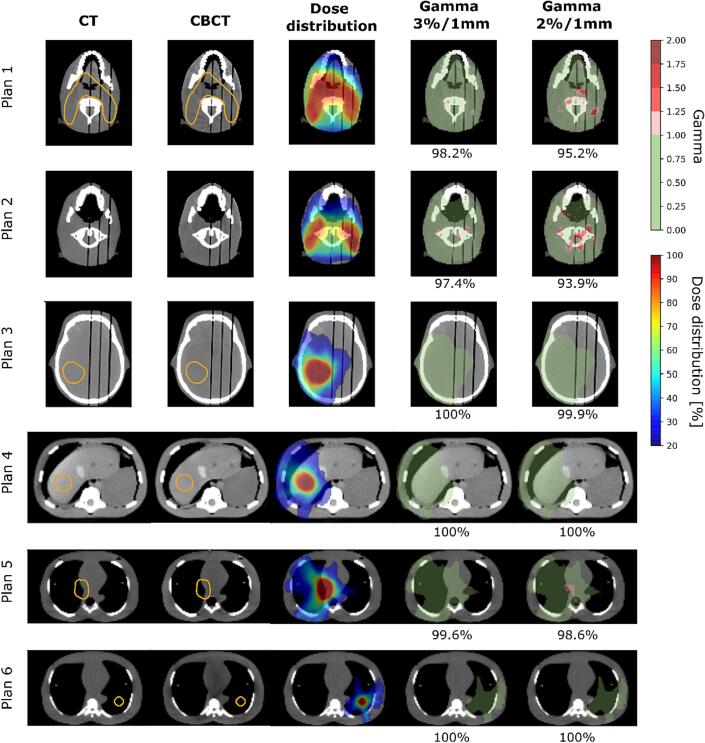
Examples of CT and CBCT images as well as corresponding planned dose distribution on the CT image and gamma maps for all investigated plans. Orange contours show the target, if present on the given slice. The target volumes for Plan 1 and Plan 2 were the same. The slice positions were chosen based on the location of the largest dose difference. Green shading on the gamma maps indicates dose regions where gamma < 1. The gamma maps show both over- and under-dosage with a red color. Dose distribution is shown with 20 % isodose as lower threshold.

Fig. 4 illustrates the CT and CBCT images, the calculated dose distribution and the gamma evaluations for each plan. The gamma pass rate for the total dose distributions at the 3 %/1mm level was 98.2 %, 97.4 %, 100 %, 100 %, 99.6 %, and 100 % for Plans 1–6, respectively. At 2 %/1mm, the gamma pass rate decreased to 93.9 % for Plan 2, but showed an agreement above 95 % for the other plans (see Fig. 4). In Plan 2, the largest discrepancies in the gamma analysis were observed for a slice superior to the cranial edge of the PTV and 3 cm from the tooth implant in the superior-inferior direction and were caused by a small rotation (0.4°) of the head phantom between the CT and CBCT scans.

## 4 Discussion

This study presented an evaluation of the HyperSight^TM^ CBCT by assessing the dose calculation accuracy compared to a fan-beam CT, as no literature has been available on this system until now. This system showed accurate CT numbers allowing direct calibration of a CT-number-to-mass-density conversion curve following the guidelines for CT as used in the clinic. We demonstrated that CT-based and CBCT-based dose distributions showed an agreement above 97 % and 93 % for gamma analysis with criteria 3 %/1mm and 2 %/1mm, respectively, and in most cases a gamma pass rate of almost 100 % was obtained for both gamma criteria, with a slight over-estimation of the CBCT-based dose calculation.

Although this assessment was phantom-based and did not incorporate patient data, it allows to estimate the performance of the CBCT-based dose calculation in the absence of anatomical changes. The head phantom consists of 4 slabs, which need to be detached in order to place tooth implants. First, all required phantom configurations were scanned on the CT and later the phantom was transferred to CBCT. Therefore, small differences in the air gaps between the phantom slabs comparing CT and CBCT scans occurred. This study only used rigid registration between the two images to not alter the internal geometry. The other phantoms did not have structural differences between CT and CBCT and rigid registration was sufficient. For patients, image registration errors may be larger [43] as anatomical changes can arise and image registration errors could influence dose differences. In this study, it is expected that the dose differences seen in here only come from differences between the two image modalities or residual phantom misalignments.

The lower dose agreement found in Plan 2 could be explained by more streak artifacts in the CBCT image due to the presence of both the metal tooth implant and the metal spine prosthesis, as well as small phantom rotation between the two scans. However, the highest deviations in D_mean_ for the OARs were still less than 1.1 % of the prescription dose and were not observed in direct proximity to the tungsten tooth implant. Compared to a previous study, the gamma pass rate of Plan 2 (97.4 %; 3 %/1mm criterion) showed better agreement than for a Varian Truebeam (Varian Medical Systems, Palo Alto, CA, USA) with a gamma pass rate of 94.5 % for a 3 %/3mm criterion in an anthropomorphic head phantom [44]. Even though the previous study reported gamma pass rate close to general clinical acceptance threshold of 95 %, it is important to emphasize that they used less strict gamma criteria and evaluated simpler planning approaches intended for palliative treatment, whereas, in this study conformal treatment planning techniques with steeper dose gradients were used. In addition, in this study, all DVH differences between CT and CBCT were below 2 %, which was also considered clinically acceptable for studies comparing dose calculation based on CT images and bulk density override in magnetic resonance imaging (MRI) [45].

In recent studies, CBCT-based dose calculation has shown less accurate results than CT due to variations in CT numbers caused by artifacts [10]. However, the degree of CT number variation depends on the CBCT system. An earlier study showed dose differences in thoracic cases of ∼ 2 % between FDK-reconstructed CBCT images acquired on a Varian C-arm linac and CT [23]. The integration of iCBCT has improved the overall performance and image quality compared to FDK reconstruction, but it was demonstrated that inconsistencies remained when dealing with different scattering conditions [4], [5], [10], [19], [33], [34]. In the previous versions of Halcyon CBCT [5], [10], larger dose differences were observed for lung phantoms in comparison to this study. In this study, the 2 %/1mm gamma passing rate for the two plans simulating lung cancer (Plan 5 and 6) was 98.6 % and 100 %. In a similar study [5], conducted on the previous version of the Halcyon platform, only 98 % agreement was observed for a gamma criterion of 2 %/2mm.

In this study, the CBCT yielded realistic CT numbers following the same trend as seen for CT (data provided in [17]), which allowed for a CT-number-to-mass-density conversion curve generated following the procedure for CT. From Table S3, it is seen that the accuracy of the CBCT and CT conversion curves are the same. Several studies have investigated methods to overcome the potential difference in CT numbers between CBCT and CT images. These studies have employed image post-processing, e.g., overriding the MDs [9], [24] or synthetic CT generation [27], [28], [29]. However, in this study such additional post-processing steps were not needed, since the CT numbers of the CBCT images were invariant, most likely due to the reduction of scatter using an anti-scatter grid in combination with an estimation of the scatter component as part of image reconstruction. These CBCT images would therefore be suitable for on-line adaptive radiotherapy workflows removing the need for synthetic CTs or registration of the planning CT image to the daily CBCT image [7], [8], [25], which improve the trust in the workflow. The time gain needs to be further evaluated given longer reconstruction time using the iterative reconstruction algorithm. Images acquired with a calibrated conversion curve would also allow for off-line adaptive workflows or CBCT only workflows for palliative cases [8]. However, before implementing CBCT-only approaches, the novel system must be evaluated on target and OAR segmentation performance in real patients, as is done for previous CBCT systems [46]. Another limitation of this work is that it does not include very large phantoms that would represent the performance on obese patients.

In conclusion, this study demonstrated comparable results between CBCT-based and CT-based dose calculation with a proper CT-number-to-mass-density conversion curve for CBCT and opens possibilities for off-line and on-line re-planning due to enhanced image quality and accurate CT numbers.

## CRediT authorship contribution statement

**Marta Bogowicz:** Conceptualization, Methodology, Software, Formal analysis, Investigation, Visualization, Writing – original draft, Writing – review & editing. **Didier Lustermans:** Writing – original draft, Visualization, Writing – review & editing. **Vicki Trier Taasti:** Writing – review & editing, Formal analysis. **Colien Hazelaar:** Writing – review & editing, Formal analysis. **Frank Verhaegen:** Funding acquisition, Writing – review & editing. **Gabriel Paiva Fonseca:** Funding acquisition, Writing – review & editing. **Wouter van Elmpt:** Supervision, Funding acquisition, Writing – review & editing.

## Declaration of competing interest

The authors declare that they have no known competing financial interests or personal relationships that could have appeared to influence the work reported in this paper.
