# Supplementary material for: Evaluation of a cone-beam computed tomography system calibrated for accurate radiotherapy dose calculation
Source: Phys Imaging Radiat Oncol. 2024 Feb 29;29:100566. doi: 10.1016/j.phro.2024.100566 (PMC10937948; doi:10.1016/j.phro.2024.100566)
Supplement: Supplementary data 1 [file mmc1.docx]

Supplementary Materials

This is the supplementary material of *“Evaluation of a cone-beam computed tomography system calibrated for accurate radiotherapy dose calculation”*.

**S.1 Target and organ-at-risk contours for the six studied plans**


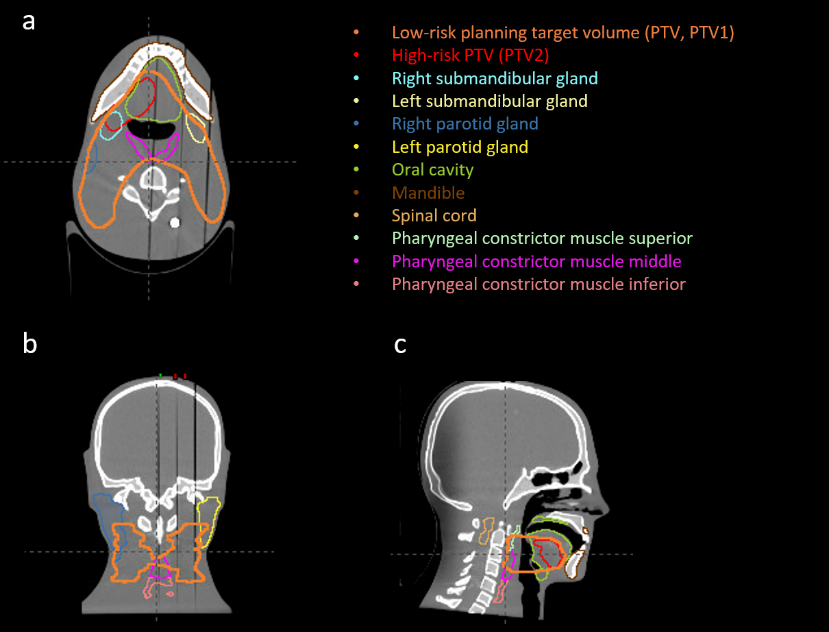


**Figure S.1:** Cross-plane views of the CT scan of Phantom 1, showing the target and organ-at-risk contours used in Plan 1 and Plan 2; a) transversal plane, b) frontal plane, c) sagittal plane. The planes are centered at the plan isocenter (gray dashed crosshair). With the contours shown in the legend.


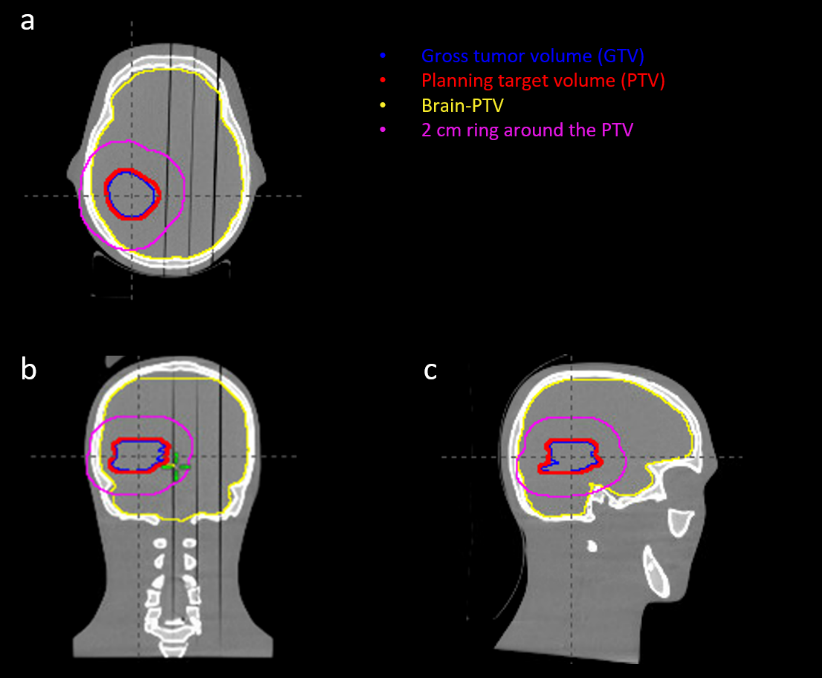


**Figure S.2:** Cross-plane views of the CT scan of Phantom 1 with the target and organ-at-risk contours used in Plan 3; a) transversal plane, b) frontal plane, c) sagittal plane. The planes are centered at the plan isocenter (gray dashed crosshair).


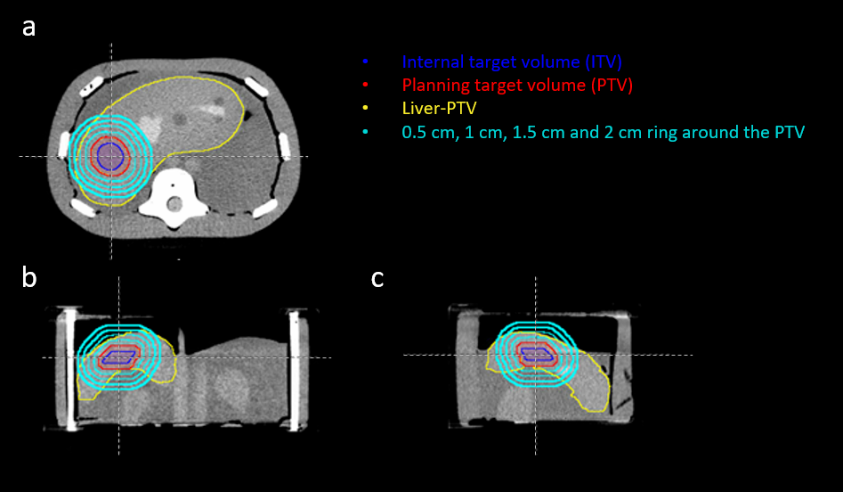


**Figure S.3:** Cross-plane views of the CT image of Phantom 2 with the target and organ-at-risk contours used in Plan 4; a) transversal plane, b) frontal plane, c) sagittal plane. The planes are centered at the plan isocenter (gray dashed crosshair). The contours are shown in the legend.


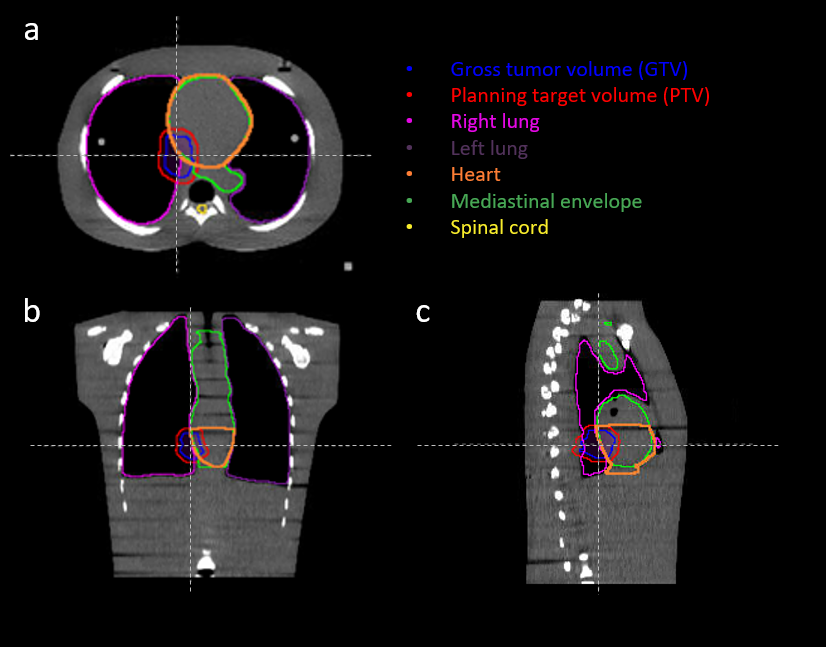


**Figure S.4:** Cross-plane views of the CT image of Phantom 3 with the target and organ–at-risk contours used in Plan 5; a) transversal plane, b) frontal plane, c) sagittal plane. The planes are centered at the plan isocenter (gray dashed crosshair).


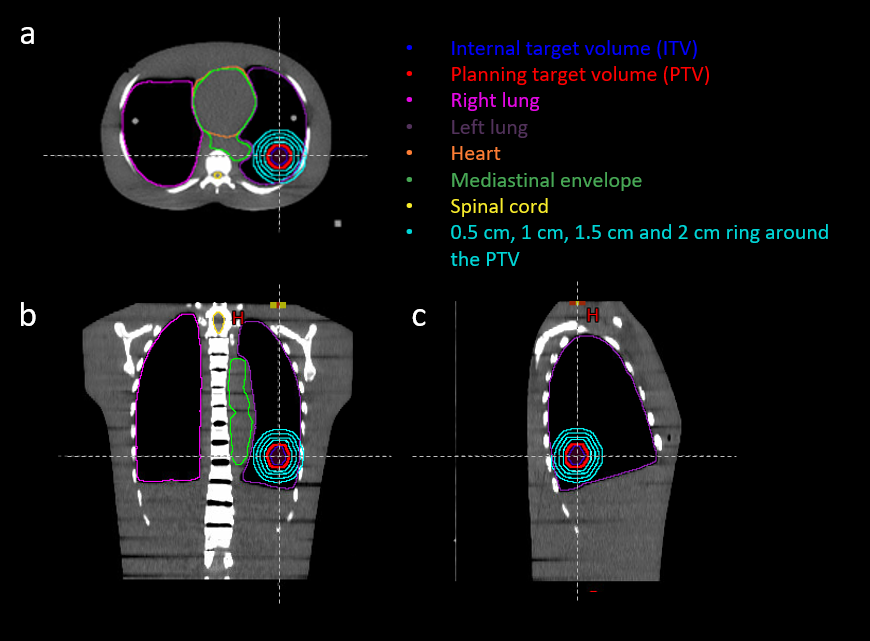


**Figure S.5:** Cross-plane views of the CT image of Phantom 3 with the target and organ-at-risk contours used in Plan 6; a) transversal plane, b) frontal plane, c) sagittal plane. The planes are centered at the plan isocenter (gray dashed crosshair).

**S.2 CT-number-to-mass-density conversion curves for CBCT**

This section will give more information about the CBCT conversion curve generation for the HyperSight^TM^ CBCT system and follows the consensus guide by Peters et al, Radiother Oncol (2023).

In Table S.1, the mean CT numbers (extracted from a volume-of-interest covering the inner 70% of the phantom insert diameter but including several slices around the middle of the phantom) used for the calibration of the conversion curves for the CBCT scans are listed. These CT numbers were used for both CT number estimation for tabulated human tissues and for the calibration of the curves themselves, as specified in the consensus guide. The conversion curves for the 125 kVp and 140 kVp are shown in Figure S.6. Separate curves were created for the small and the large phantom size, as well as for the 125 kVp and 140 kVp spectra, to account for the dependency of CT numbers on both phantom size and energy spectrum, with more variations in the high-density materials (see Table S.1). The connection points between the specified tissue groups (lung, adipose, soft tissue and bone) are indicated for the four curves in Table S.2. More details can be found in the paper by Peters et al, Radiother Oncol (2023).

In this study, the same procedure was used as in clinical practice, which is to use the small phantom conversion curve for brain and head-and-neck patients and the large phantom conversion curve for all other treatment sites. Therefore, it was in this study chosen to use the small phantom conversion curve for phantom 1 (Plan 1, 2 and 3) and the large phantom conversion curve for phantom 2 and 3 (Plan 4, 5 and 6).

**Table S.1**: Mass densities (specified batch-specific by phantom vendor), relative electron densities (not used in the current study), and measured CT numbers for phantom inserts of the Gammex Advanced Electron Density Phantom used for calibration of CT-number-to-mass-density conversion curves.

| **Insert name** | **Mass density (g/cm^3^)** | **Relative electron density** | **125 kVp** | | **140 kVp** | |
| --- | --- | --- | --- | --- | --- | --- |
|  |  |  | **Small phantom** | **Large phantom** | **Small phantom** | **Large phantom** |
| LN300 | 0.290 | 0.280 | -735 | -723 | -731 | -722 |
| LN450 | 0.480 | 0.463 | -545 | -524 | -541 | -516 |
| HE Adipose | 0.961 | 0.948 | -81 | -78 | -78 | -89 |
| HE Breast | 0.986 | 0.970 | -56 | -44 | -53 | -43 |
| Liquid Water | 1.000 | 1.000 | -7 | -4 | -5 | 2 |
| Solid Water | 1.022 | 0.996 | -8 | -1 | -6 | -15 |
| HE Brain | 1.051 | 1.023 | 29 | 34 | 30 | 39 |
| HE Liver | 1.082 | 1.053 | 57 | 67 | 58 | 58 |
| HE Inner Bone | 1.207 | 1.154 | 343 | 316 | 329 | 289 |
| CB30% | 1.330 | 1.263 | 526 | 482 | 506 | 453 |
| CB50% | 1.557 | 1.457 | 961 | 888 | 919 | 846 |
| Cortical Bone | 1.923 | 1.683 | 1445 | 1292 | 1376 | 1232 |
| HE Cortical Bone | 1.822 | 1.770 | 1593 | 1418 | 1516 | 1359 |


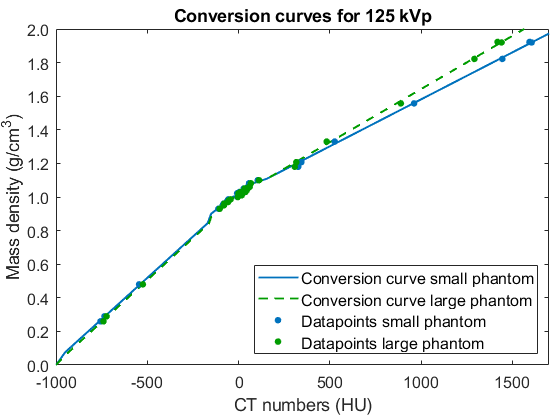

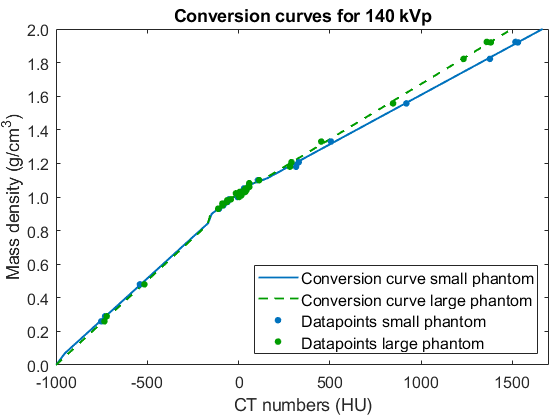


**Figure S.6:** CT-number-to-mass-density conversion curves for 125 kVp (left) and 140 kVp (right) generated for the small phantom (blue line) and large phantom (green dashed line).

**Table S.2**: Connection points for the four conversion curves for the CBCT system, following the recommendation given in Peters et al, Radiother Oncol (2023). Note, the last point at a CT number of 2000 HU for each curve is not a connection point but an extrapolation point, meaning that the curves do not change slope at this point.

| **Small 125 kVp** | | **Large 125 kVp** | | **Small 140 kVp** | | **Large 140 kVp** | |
| --- | --- | --- | --- | --- | --- | --- | --- |
| CT number | Mass density (g/cm^3^) | CT number | Mass density (g/cm^3^) | CT number | Mass density (g/cm^3^) | CT number | Mass density (g/cm^3^) |
| -1024 | 0.0012 | -1024 | 0.0012 | -1024 | 0.0012 | -1024 | 0.0012 |
| -999 | 0.0012 | -999 | 0.0012 | -999 | 0.0012 | -999 | 0.0012 |
| -950 | 0.0757 | -950 | 0.0573 | -950 | 0.0711 | -950 | 0.0505 |
| -170 | 0.8434 | -163 | 0.8411 | -167 | 0.8441 | -172 | 0.8327 |
| -150 | 0.9009 | -143 | 0.9013 | -147 | 0.8998 | -152 | 0.9025 |
| -30 | 1.0012 | -30 | 0.9953 | -30 | 0.9987 | -30 | 1.0000 |
| 0 | 1.0108 | 0 | 1.0035 | 0 | 1.0089 | 0 | 1.0056 |
| 67 | 1.0767 | 77 | 1.0802 | 68 | 1.0761 | 69 | 1.0750 |
| 155 | 1.1088 | 162 | 1.1123 | 156 | 1.1109 | 163 | 1.1226 |
| 2000 | 2.1410 | 2000 | 2.2759 | 2000 | 2.1977 | 2000 | 2.3307 |


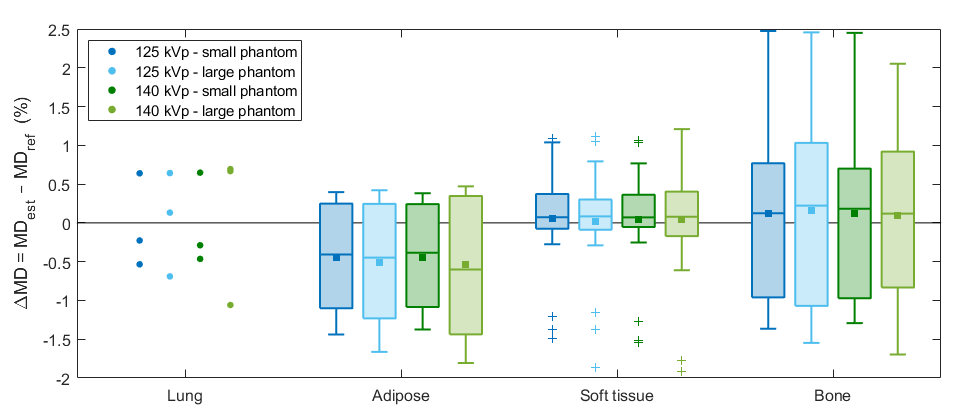


**Figure S.7:** Accuracy of the four conversion curves for the CBCT, based on the datapoints (phantom inserts and tabulated human tissues) used to generate the curves for each of the four tissue groups (lung, adipose, soft tissue and bone; each tissue group specifying one curve segment of the piecewise linear curves). Due to the low number of lung tissues (N=3, phantom inserts and tabulated human tissues), these datapoints are shown individually, while boxplots are shown for the other three tissue groups. For the boxplots, the boxes represent the 25%-percentile to the 75%-percentile, with the horizontal line within the boxes representing the median, and the square representing the mean. The whiskers extend to 1.5 times the inter-quartile range, and datapoints outside this range are seen as outliers and shown with +-symbols.

**Table S.3:** Accuracy of the four CT-number-to-mass-density conversion curves for the CBCT and the two curves for CT. In contrast to Figure S.7, the metrics are here given over all the datapoints included in the curve fitting, and not per tissue group. *Abbreviation*: RMSE – root-mean-square error.

|  | **125 kVp** | | **140 kVp** | | **CT 120 kVp** | |
| --- | --- | --- | --- | --- | --- | --- |
|  | **Small** | **Large** | **Small** | **Large** | **Small** | **Large** |
| Mean error (%) | -0.1% | -0.1% | -0.1% | -0.1% | -0.1% | -0.1% |
| Mean absolute error (%) | 0.6% | 0.6% | 0.6% | 0.7% | 0.6% | 0.6% |
| RMSE (%) | 0.8% | 0.9% | 0.8% | 0.9% | 0.8% | 0.8% |

**S.3 Dose calculation accuracy**


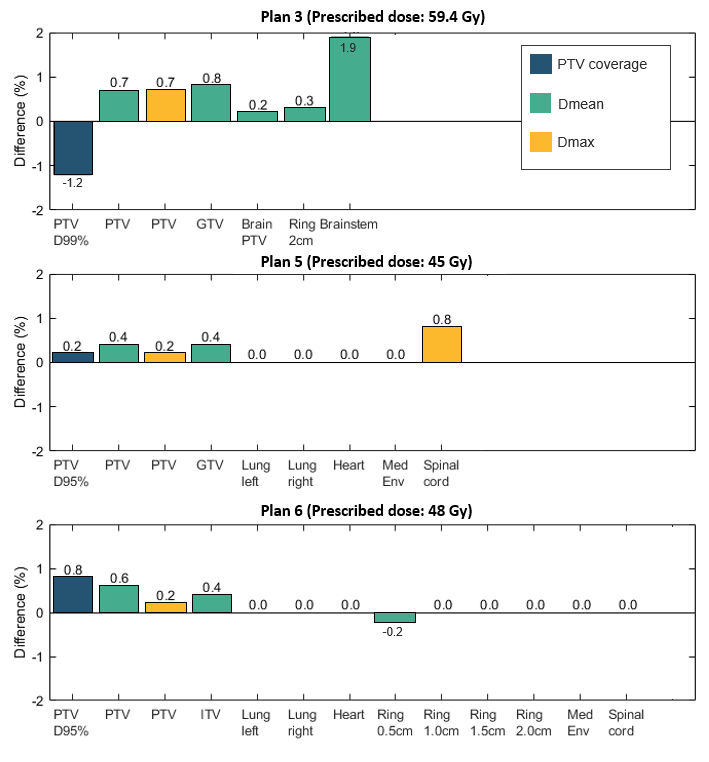


**Figure S.8:** Bar plots for dose differences (∆D = D_CBCT_ – D_CT_; given as percentage of prescribed dose) for the evaluated dose-volume-histogram (DVH) parameters between dose calculation on fan-beam CT and CBCT for Plan 3, 5 and 6. The planning target volume (PTV) coverage (blue bars) is quantified by D_99%_ (Plan 3) and D_95%_ (Plan 5 and 6). (The results for Plan 1, 2, and 4 can be seen in Figure 3 in the main text). *Abbreviations:* Med Env – Mediastinal Envelope.

**Table S.4:** A detailed description of the differences in dose-volume-histogram (DVH) parameters (in Gy (column 5) and percentage of prescribed dose (last column)) between CT and CBCT dose calculations. The DVH metrics indicate the target coverage, Dmean and Dmax (given by D0.03cc) differences for the planning target volume (PTV) and organs-at-risk (OARs) for each individual plan. (The table continues on the next pages).

| **Plan 1** |  |  |  |  |  |
| --- | --- | --- | --- | --- | --- |
| **DVH metric** | **Region** | **CT [Gy]** | **CBCT [Gy]** | **Difference CBCT – CT [Gy]** | **Difference CT – CBCT [%]** |
| Coverage | PTV1 D98% | 51.3 | 51 | -0.3 | 0.4 |
|  | PTV2 D98% | 66.5 | 66.9 | 0.4 | 0.6 |
| Dmean | PTV1 | 57.2 | 57.6 | 0.4 | 0.6 |
|  | PTV2 | 70.7 | 71.2 | 0.5 | 0.7 |
|  | Parotid_L | 18.6 | 18.5 | -0.1 | 0.1 |
|  | Parotid_R | 24 | 24.5 | 0.5 | 0.7 |
|  | Oral_cavity | 50.1 | 50.4 | 0.3 | 0.4 |
|  | PCM_Inf | 10.2 | 10.3 | 0.1 | 0.1 |
|  | PCM_Med | 52.8 | 53.3 | 0.5 | 0.7 |
|  | PCM_Sup | 55.4 | 55.8 | 0.4 | 0.6 |
|  | Submandibular_L | 45.5 | 45.9 | 0.4 | 0.6 |
|  | Submandibular_R | 64.2 | 64.6 | 0.4 | 0.6 |
| Dmax | PTV2 | 74.3 | 74.8 | 0.5 | 0.7 |
|  | Spinal cord | 41.2 | 41.4 | 0.2 | 0.3 |
| **Plan 2** |  |  |  |  |  |
| **DVH metric** | **Region** | **CT [Gy]** | **CBCT [Gy]** | **Difference CBCT – CT [Gy]** | **Difference CT – CBCT [%]** |
| Coverage | PTV1 D98% | 52.3 | 52.2 | -0.1 | 0.1 |
|  | PTV2 D98% | 66.5 | 66.3 | -0.2 | 0.3 |
| Dmean | PTV1 | 59 | 59.1 | 0.1 | 0.1 |
|  | PTV2 | 71.3 | 71.8 | 0.5 | 0.7 |
|  | Parotid_L | 19.6 | 19 | -0.6 | 0.9 |
|  | Parotid_R | 25 | 25.4 | 0.4 | 0.6 |
|  | Oral_cavity | 50.8 | 50.9 | 0.1 | 0.1 |
|  | PCM_Inf | 12.8 | 13.3 | 0.5 | 0.7 |
|  | PCM_Med | 55.1 | 55.9 | 0.8 | 1.1 |
|  | PCM_Sup | 55.0 | 55.2 | 0.2 | 0.2 |
|  | Submandibular_L | 48 | 48.2 | 0.2 | 0.3 |
|  | Submandibular_R | 65.2 | 65.2 | 0 | 0.0 |
| Dmax | PTV2 | 75.6 | 76.7 | 1.1 | 1.5 |
|  | Spinal cord | 40.8 | 41.8 | 1.0 | 1.4 |
| **Plan 3** |  |  |  |  |  |
| **DVH metric** | **Region** | **CT [Gy]** | **CBCT [Gy]** | **Difference CBCT – CT [Gy]** | **Difference CT – CBCT [%]** |
| Coverage | PTV D99% | 56.7 | 56 | -0.7 | 1.2 |
| Dmean | GTV | 61.1 | 61.6 | 0.5 | 0.8 |
|  | PTV | 60.8 | 61.2 | 0.4 | 0.7 |
|  | Brain-PTV | 8.3 | 8.4 | 0.1 | 0.2 |
|  | Ring_2cm | 23.7 | 23.9 | 0.2 | 0.3 |
| Dmax | PTV | 64.6 | 65.0 | 0.4 | 0.7 |
|  | Brianstem | 37.0 | 38.2 | 1.1 | 1.9 |
| **Plan 4** |  |  |  |  |  |
| **DVH metric** | **Region** | **CT [Gy]** | **CBCT [Gy]** | **Difference CBCT – CT [Gy]** | **Difference CT – CBCT [%]** |
| coverage | PTV D99% | 60 | 60.3 | 0.3 | 0.5 |
| Dmean | ITV | 68.8 | 68.9 | 0.1 | 0.2 |
|  | PTV | 66.1 | 66.3 | 0.2 | 0.3 |
|  | Liver-PTV | 10.4 | 10.4 | 0 | 0.0 |
|  | Ring_0.5cm | 52.1 | 52.7 | 0.6 | 1.0 |
|  | Ring_1cm | 33.3 | 33.6 | 0.3 | 0.5 |
|  | Ring_1.5cm | 19.7 | 19.8 | 0.1 | 0.2 |
|  | Ring_2cm | 14.2 | 14.3 | 0.1 | 0.2 |
| Dmax | PTV | 70.9 | 71.2 | 0.4 | 0.7 |
| **Plan 5** |  |  |  |  |  |
| **DVH metric** | **Region** | **CT [Gy]** | **CBCT [Gy]** | **Difference CBCT – CT [Gy]** | **Difference CT – CBCT [%]** |
| Coverage | PTV D95% | 42.8 | 42.9 | 0.1 | 0.2 |
| Dmean | GTV | 45.2 | 45.4 | 0.2 | 0.4 |
|  | PTV | 44.9 | 45.1 | 0.2 | 0.4 |
|  | Lung_L | 2.5 | 2.5 | 0 | 0.0 |
|  | Lung_R | 8.0 | 8.0 | 0 | 0.0 |
|  | Heart | 14.5 | 14.5 | 0 | 0.0 |
| Dmax | PTV | 47.8 | 47.9 | 0.1 | 0.2 |
|  | Mediastinal envelope | 47.7 | 47.7 | 0 | 0 |
|  | Spinal cord | 10.2 | 10.6 | 0.3 | 0.8 |
| **Plan 6** |  |  |  |  |  |
| **DVH metric** | **Region** | **CT [Gy]** | **CBCT [Gy]** | **Difference CBCT – CT [Gy]** | **Difference CT – CBCT [%]** |
| Coverage | PTV D95% | 48 | 48.4 | 0.4 | 0.8 |
| Dmean | ITV | 62.1 | 62.3 | 0.2 | 0.4 |
|  | PTV | 56.3 | 56.6 | 0.3 | 0.6 |
|  | Lung_L | 6.2 | 6.2 | 0 | 0.0 |
|  | Lung_R | 0.9 | 0.9 | 0 | 0.0 |
|  | Heart | 1.8 | 1.8 | 0 | 0.0 |
|  | Ring_0.5cm | 37.1 | 37 | -0.1 | 0.2 |
|  | Ring_1cm | 22.1 | 22.1 | 0 | 0.0 |
|  | Ring_1.5cm | 14.3 | 14.3 | 0 | 0.0 |
|  | Ring_2cm | 10.1 | 10.1 | 0 | 0.0 |
| Dmax | PTV | 65.4 | 65.5 | 0.1 | 0.2 |
|  | Mediastinal envelope | 16.8 | 16.8 | 0.0 | 0.0 |
|  | Spinal cord | 7.3 | 7.3 | 0.0 | 0.0 |
